# Real World Management of Anaphylaxis Versus the National Institute for Health and Clinical Excellence (NICE) Guidelines

**DOI:** 10.7759/cureus.29336

**Published:** 2022-09-19

**Authors:** Iman Nasr, Asmaa S Mahdi, Jalila Al Shekaili, Ikram Nasr, Humaid Al Wahshi, Saad Al Juma, Latifa Al Shukeili, Ibrahim Al Zakwani, Issa Al Salmi

**Affiliations:** 1 Allergy and Immunology, The Royal Hospital, Muscat, OMN; 2 Allergy and Immunology, Sultan Qaboos University Hospital, Seeb, OMN; 3 Gastroenterology, Guy's and St Thomas' NHS Foundation Trust, London, GBR; 4 Rheumatology, The Royal Hospital, Muscat, OMN; 5 Emergency Department, The Royal Hospital, Muscat, OMN; 6 Allergy and Immunology, Al Nahdah Hospital, Muscat, OMN; 7 Pharmacy, Sultan Qaboos University Hospital, Seeb, OMN; 8 Nephrology, The Royal Hospital, Muscat, OMN

**Keywords:** oman, nice guidelines, adrenaline auto-injector, anaphylaxis, angioedema, urticaria

## Abstract

Objectives

Anaphylaxis is an acute, life-threatening immediate allergic reaction caused by the sudden systemic release of mediators from mast cells. This study aims to assess the current practice of emergency management of children and adults diagnosed with anaphylaxis at the Royal Hospital, Muscat, Oman, in line with the National Institute for Health and Clinical Excellence (NICE) guidelines.

Methods

This is an observational retrospective study of all anaphylaxis cases seen at the emergency department (ED) from January 2013 to January 2018 and compared with the management of anaphylaxis in the ED as per the NICE guidelines. Inclusion criteria were all patients, children (age 16 and below), and adults diagnosed with anaphylaxis based on the World Allergy Organization (WAO) criteria. Exclusion criteria are all cases labeled as anaphylaxis that did not match the WAO criteria for anaphylaxis.

Results

Of 100 patients with a preliminary diagnosis of anaphylaxis, 49 patients (49%) were true-anaphylaxis cases based on the WAO definition 16 were children (age 16 years and below), and 33 were adults ( age 16 years and above). The other 51 patients (51%) with misdiagnosed anaphylaxis were later diagnosed with spontaneous urticaria, septic shock, vocal cord dysfunction, severe asthma, and anxiety attack. All 49 patients with true-anaphylaxis appropriately received adrenaline intramuscularly at the ED. All 16 children were admitted, seen by an allergist, and received an adrenaline auto-injector when indicated. Only 5 of the 33 adults were admitted and seen by an allergist, and 4 of those required an adrenaline auto-injector upon discharge. The remaining 28 adults were discharged from the ED, and only 3 of these were referred to the allergist. None received an adrenaline auto-injector upon discharge from the ED, and no mention in the ED notes on patient education regarding allergen avoidance.

Conclusion

Third of the patients who presented to ED were children (<16 years), and two third were adults. Insect venom was the main reason for anaphylaxis in both age groups. There was an underutilization of adrenaline auto-injector prescriptions for adult patients. This could be very well improved by disseminating policies and guidelines to adult physicians.

## Introduction

Anaphylaxis is a severe, life-threatening, generalized type I hypersensitivity reaction, if not diagnosed and managed early, can lead to fatal or near-fatal outcomes. It is an acute systemic IgE-mediated allergic response caused by the sudden release of mediators, including histamine, tryptase, cytokines, leukotrienes, and prostaglandins from mast cells. These mediators give rise to cardiovascular, respiratory, and skin symptoms, among others. Such a patient would typically present with breathing difficulty or circulatory compromise (hypotension or tachycardia), among other symptoms [1-3]. Mast cell tryptase is a marker of mast cell activation and degranulation. It can help confirm the diagnosis of anaphylaxis in cases where the diagnosis of anaphylaxis is questionable (anaphylaxis mimickers). Mimickers of anaphylaxis include acute exacerbation of asthma, vocal cord dysfunction, vasovagal syncope, acute anxiety, panic attack, and septic shock. A normal tryptase level does not exclude anaphylaxis [1-3].

The incidence of anaphylaxis varies between 0.05-2% [4]. Manifestations of patients with anaphylaxis are variable. They include all or a combination of the following; cutaneous such as pruritus and urticaria, mucosal including rhinitis and conjunctivitis, gastrointestinal manifestations such as vomiting, respiratory like dyspnea, cardiovascular such as hypotension, and neurological such as loss of consciousness [5,6]. Overall, the most common triggers of anaphylaxis are foods, insect venom, and medication allergy [6-8].

The primary prevention of anaphylaxis is through strict avoidance of the allergen. Diagnosis of anaphylaxis may be difficult without a clear history of allergy and in the absence of symptoms from the skin and mucosal tissue. Adrenaline is the only evidence-based first-line treatment of anaphylaxis [9]. Adrenaline should be administered acutely on presentation and prescribed for the patient until seeing an allergist for a proper evaluation and consultation. Early recognition of the signs and symptoms of anaphylaxis, along with prompt administration of intramuscular adrenaline (epinephrine), especially in the ED, is crucial in successfully managing anaphylaxis [10].

Despite the wide availability of international guidelines on the recognition and management of allergy in general and anaphylaxis in specific, anaphylaxis is underdiagnosed worldwide and undertreated [3,7,11]. In a multicenter study in the USA, only 24% of those with severe allergic reactions received epinephrine at the ED, and only 16% of those patients were given self-injectable epinephrine. Furthermore, only 12% were subsequently referred to specialty allergy care [12]. In another study in Australia, the use of adrenaline was just under 40% in ED [6].

In our study, we assessed the current practice in the care of adults and children with anaphylaxis presenting to the emergency department (ED) at the Royal Hospital, Muscat, Oman, against the recommendations in the NICE guidelines (Table 2) over five years from January 2013 to January 2018.

## Materials and methods

This is a retrospective observational study performed at the Royal Hospital Emergency Department and collecting data for a 5-years period from January 2013 to January 2018. All clinical data were collected retrospectively from the computerized medical records system called Al Shifa. The study was approved by the local Scientific Ethical Committee at The Royal Hospital (RH) Ref. No. 60/2018. We defined anaphylaxis based on the World Allergy Organization (WAO) definition (Table 1). Inclusion criteria included all patients diagnosed with anaphylaxis based on the World Allergy Organization (WAO) definition. Exclusion criteria are all cases labeled as anaphylaxis; however, they did not match the WAO definition. We compared the management of anaphylaxis in the ED between adults and children against the recommendations by the NICE guidelines (Table 2). Moreover, we assessed the clinical presentation and triggers in both groups.

**Table 1:** WAO clinical criteria for the diagnosis of anaphylaxis, NIAID/FAAN) symposium, 2005: PEF- peak expiratory flow

Anaphylaxis is diagnosed if any one of the 3 criteria is met:
1. Acute onset of an illness (minutes to several hours) with involvement of the skin, mucosa, or both (e.g., generalized hives, pruritus or flushing, swollen lips-tongue-uvula), PLUS AT LEAST ONE OF THE FOLLOWING:
a) Respiratory compromise (e.g., dyspnea, wheeze-bronchospasm, stridor, reduced PEF, hypoxemia)
b) Reduced BP or associated symptoms of end-organ dysfunction (e.g., hypotonia (collapse, syncope, incontinence)
2. Two or more of the following that occurs rapidly after exposure to a likely allergen for that patient(minutes to several hours):
a) Involvement of the skin-mucosal tissue (e.g., generalized hives, itch-flush, swollen lips-tongue-uvula)
b) Respiratory compromise (e.g., dyspnea, wheeze-bronchospasm, stridor, reduced PEF, hypoxemia)
c) Reduced BP or associated symptoms (e.g., hypotonia [collapse], syncope, incontinence)
d) Persistent gastrointestinal symptoms (e.g., crampy abdominal pain, vomiting)
3. Reduced BP after exposure to known allergen for that patient (minutes to several hours):
• Infants and children: low systolic BP (age specific) or greater than 30% decrease in systolic BP
• Adults: systolic BP of less than 90 mm Hg or greater than 30% decrease from that person's baseline
• Low systolic blood pressure for children is defined as less than 70 mm Hg from 1 month to 1 year, less than (70 mm Hg + [2 × age]) from 1 to 10 years, and less than 90 mm Hg from 11 to 17 years

**Table 2:** The NICE guidelines for the treatment of anaphylaxis NICE- National Institute for Health and Clinical Excellence; MCT- mast cell tryptase (MCT)

1. The clinical features should be documented.
2. The time of onset of the reaction should be recorded.
3. The circumstances immediately before the onset of symptoms to identify triggers.
4. Timed blood samples for mast cell tryptase (MCT) testing should be taken in suspected anaphylaxis from patients 16 years or above.
5. A second MCT within 1–2 hours (but no later than 4 hours) from the onset of symptoms must be taken.
6. Children < 16, are to be admitted to the hospital under the care of a pediatric medical team.
7. The patient should be offered a referral to a specialist allergy service.
8. After emergency treatment for suspected anaphylaxis people should be offered an adrenaline injector as an interim measure until a specialist allergy appointment.
9. Information about anaphylaxis, including the signs and symptoms of an anaphylactic reaction.
10. Information about the risk of a biphasic reaction.
11. Give information on what to do if an anaphylactic reaction occurs (use the adrenaline injector and call emergency services.
12. Demonstrate the correct use of the adrenaline injector and when to use it.
13. Give advice about how to avoid the suspected trigger (if known).
14. Provide information about the need for referral to a specialist allergy service and the referral process.
15. Provide information about patient support groups.

Statistical analyses

Qualitative data were expressed as frequency and percentage. Pearson’s Chi-square test or Fisher’s exact test was used to examine the relationship between qualitative variables. Comparison between the two groups was made using the Mann-Whitney test (non-parametric t-test) for not normally distributed quantitative data. A p-value < 0.05 was considered significant. All statistical calculations were performed using the STATA 13 software (version 13.0, STATA Inc, Chicago, Illinois, USA).

## Results

During the study period, 100 patients presented with a presumed diagnosis of anaphylaxis, of which 49 (49%) were considered true anaphylaxis cases based on the WAO criteria (table 1). The rest did not fulfill the WAO criteria as they were diagnosed based on shortness of breath alone or severe urticaria without respiratory or cardiovascular involvement. Of the 49 who fitted the diagnosis of anaphylaxis, 33 (67.3%) were adults, 15 females and 18 males with an age range of 17-66 years (median age of 33 years). Sixteen patients (32.6%) were children; seven females and nine males with an age range of 1-16 years (median age of 11 years).

All 49 patients received adrenaline intramuscularly upon their presentation to ED. The adrenaline dose was 0.5mg of 1:1000 for adults and 0.01mg/kg of 1:1000 for children. All 16 children were admitted, seen by an allergist, and received an adrenaline auto-injector when indicated. Of the 33 adults with anaphylaxis, only five were admitted as they required more than one dose of adrenaline. An allergist saw those during admission, and four required an adrenaline auto-injector upon discharge, one for the diagnosis of idiopathic anaphylaxis and three due to Pachycondyla Sennaarensis (black ant, locally known as Samsum) anaphylaxis (table 3, 4). Laboratory tests in ED after stabilizing the patients included complete blood count and kidney and liver functions but no tryptase, which would have been helpful in the case diagnosed as idiopathic anaphylaxis.

**Table 3:** Assessing the management of Anaphylaxis in the ED at Royal Hospital, Oman for both Pediatrics and Adults against the NICE Guidelines. MCT- mast cell tryptase; NICE- National Institute for Health and Clinical Excellence

Standards	Pediatrics (n=16)	Adults (n=33)	Both=49
1. The clinical features should be documented	16 (100%)	33 (100%)	49 (100%)
2. The time of onset of the reaction should be recorded	7 (42.9%)	0 (0%)	7 (14.28%)
3. The circumstances immediately before the onset of symptoms to identify trigger	16 (100%)	33 (100%)	49 (100%)
4. Timed blood samples for mast cell tryptase (MCT) testing should be taken in suspected anaphylaxis from patients 16 years or above.	0 (0%)	0 (0%)	0 (0%)
5. A second MCT within 1–2 hours (but no later than 4 hours) from the onset of symptoms must be taken	0 (0%)	0 (0%)	0 (0%)
6. Children < 16 to be admitted to the hospital under the care of a pediatric medical team	16 (100%)	NA	16 (32.65%)
7. The patient should be offered a referral to a specialist allergy service	16 (100%)	8 (24.2%)	24 (48.98%)
8. After emergency treatment for suspected anaphylaxis people should be offered an adrenaline injector as an interim measure until a specialist allergy appointment	16 (100%)	0 (0%)	16 (32.65%)
9. Information about anaphylaxis, including the signs and symptoms of an anaphylactic reaction	16 (100%) during admission	33 (100%)	49 (100%)
10. Information about the risk of a biphasic reaction.	0 (0%)	0 (0%)	0 (0%)
11. Give information on what to do if an anaphylactic reaction occurs (use the adrenaline injector and call emergency services)	16 (100%)	0 (0%)	16 (32.65%)
12. Demonstrate the correct use of the adrenaline injector and when to use it	16 (100%)	7 (21.1%)	23 (46.94%)
13. Give advice about how to avoid the suspected trigger (if known)	16 (100%)	8 (24.2%)	24 (48.98%)
14. Provide information about the need for referral to a specialist allergy service and the referral process	16 (100%)	8 (24.2%)	24 (48.98%)
15. Provide information about patient support groups	0 (0%)	0 (0%)	0 (0%)

**Table 4:** The clinical presentation of Anaphylaxis upon arrival to the ED for both Adults and Children

Symptom	Adults, n= 33 (%)	Pediatric, n=16 (%)	P Value
Angioedema, Urticaria, and Shortness of breath	18 (54.54%)	3 (18.75%)	0.005
Urticaria and Shortness of breath	14 (42.42%)	12 (75%)	0.046
Angioedema alone (laryngeal edema)	1 (3%)	1 (6.25%)	0.412

The remaining 28 adults were discharged from the ED, and only three of these were referred to the allergist. None received an adrenaline auto-injector upon discharge from the ED, and no mention in the ED notes on patient education regarding allergen avoidance.

Anaphylaxis triggers for adults were insect bite (45.45%), medications (27.3%), food (24.24%) and unknown in 3% (table 5). There was no documentation on allergen avoidance counseling by the ED physician. Triggers in children included insect bite (68.75%), medications (18.75%) and food (12.5%) (table 5). In contrast to the finding with an adult, there was appropriate counseling on allergen avoidance for all the admitted children and their parents that was provided by the immunologist and the dietitian in case of food allergy.

**Table 5:** Causes of Anaphylaxis in Adults (age >16) and Children

Triggers	Adults n= 33 (%)	Pediatric n=16 (%)	P value
Insect bite	15 (45.45%)	11 (68.75%)	0.053
Medication	9 (27.27%)	3 (18.75%)	o.065
Food (seafood and nuts)	8 (24.24%)	2 (12.5%)	0.003
Unknown	1 (3%)	0 (0%)	0.325

## Discussion

This is the first study to evaluate our center’s real-world life data on anaphylaxis diagnosis and management. The current study showed that two third of all presenting patients were adults, and the remaining patients were children, with an almost equal gender distribution. 80-90% of children with anaphylaxis have cutaneous manifestations such as urticaria, pruritus, angioedema, and flushing [5]. Other severe and red flag symptoms involving respiratory and cardiovascular systems occur in children with anaphylaxis in 60-70% and 10-30%, respectively [5,6]. The clinical presentation of the children and adults presenting to our ED is summarized in Table 4, with the majority of adults (18 out of 33, 54.5%) presented with urticaria, angioedema, and shortness of breath, while the majority of children (12 out of 16, 75%) presented with urticaria and shortness of breath.

Studies have shown the predominance of food as the main anaphylaxis trigger in children [6,8], while it was drugs followed by food in adults [6] and insects in other studies [7,8]. In contrast, at our center, the most common trigger of anaphylaxis in adults was insect bite (including black ant, bee, wasp, and unknown) (45.4%) and the same in children (68.7%). Black is widely prevalent in Oman, even indoors, with an increased risk of ant bites. Slippers and sandals are commonly worn due to the hot weather, increasing the risk of black ant bites and anaphylaxis.

 A useful multicentre study done by Clark S et al. illustrated the management of anaphylaxis secondary to food allergy in 21 ED centers in North America [12]. An average of 40% of patients were instructed to avoid the offending allergen. Only 16% were prescribed an adrenaline auto-injector on discharge from ED, and 12% were referred to an allergist. 

Mast cell tryptase is a marker of mast cell activation and degranulation. It can help confirm the diagnosis of anaphylaxis in cases where the diagnosis of anaphylaxis is questionable (anaphylaxis mimickers) [13]. This was not done in both groups due to a lack of awareness and knowledge of this test. In addition, this test is not universally available and is not specific for anaphylaxis. Other conditions where serum tryptase may be elevated are systemic mastocytosis, myocardial infarction, and trauma [13]. Serum tryptase measurement during an anaphylactic episode and another after recovery (baseline level) is more useful than performing one measurement during anaphylaxis as it is reported to improve sensitivity and specificity [13].

The mainstay of anaphylaxis management is the immediate administration of intramuscular adrenaline on presentation to the ED. All the children were admitted and referred to the immunologist in line with the NICE guidelines, which mandate that all children aged 16 years and under presenting with anaphylaxis should be admitted. This gave them the advantage of being reviewed by the immunologist and counseled on allergen avoidance and adrenaline auto-injector use when indicated prior to discharge. On the contrary, only 15% of adults were admitted after ED encounters. Most adults were discharged from the ED and were not prescribed an adrenaline auto-injector which should have been given until seen by an allergist. Moreover, there was no documentation on patient education regarding allergen avoidance in all cases from the ED. This suboptimal care was reported in other studies [13,14].

Despite many international guidelines published in the last 10 years regarding the management of anaphylaxis, it continues to be underdiagnosed, under notified and undertreated. In general, emergency department anaphylactic reactions in adults and children are frequently not recognized. They may be confused with other conditions, such as an asthmatic attack or sepsis, leading to underutilization of adrenaline and a higher risk of death [15,16]. Additionally, those correctly identified as anaphylaxis often do not receive appropriate post-anaphylaxis care [16].

Since allergen avoidance is the primary key to anaphylaxis prevention, a strong emphasis on educating patients about trigger avoidance is necessary. Anaphylaxis is most frequently seen by first-line medical staff, including emergency doctors and nurses, than allergists or immunologists [16]. Raising the awareness of the ED staff on anaphylaxis diagnosis and management is crucial to improve the management of this severe and possibly fatal condition [17].

In our center, the Adult Allergy and Immunology Unit developed local guidelines for managing anaphylaxis for adults due to the significant gaps in management. Regular teachings were provided to the ED staff by senior ED consultants. Therefore, all patients with anaphylaxis were referred to the allergist/immunologist, whether admitted or discharged. This will be followed by re-auditing the cases diagnosed with anaphylaxis admitted to the ED.

The limitations of this study include the small number of patients due to the rarity of the condition and the fact that the data is assessed from one center. Moreover, being a retrospective study means a lack of specific information such as the onset of reaction, the time of adrenaline administration, and whether the patient was educated on allergen avoidance, among others. Another limitation is that the inclusion criteria was a presumed diagnosis of anaphylaxis, and cases diagnosed as ‘allergy’ or ‘allergic reaction’ were not looked at. Anaphylaxis may have been the diagnosis in some of these cases, unrecognized or underdiagnosed.

## Conclusions

The pediatric allergy service at the Royal Hospital, Oman, was established in 2012 which led to the proper education of the pediatric emergency staff on the management of anaphylaxis. In contrast, the adult allergy service was only established in 2016. After this study, it was very clear that work was needed to spread awareness on the management of anaphylaxis and aftercare through the creation of a local guideline and a series of lectures focusing on the management of anaphylaxis as well as educating the ED staff on the aftercare of patients who suffered anaphylaxis and do not require admission mainly allergen avoidance and prescription of an adrenaline auto-injector on discharge until seen by an allergist/immunologist since accidental re-exposure to the allergen such as an insect bite can be fatal. Emphasis was made that all patients with anaphylaxis should be referred to the allergist/immunologist, whether admitted or discharged. A re-audit is required to ensure that practice standards are ongoing.
